# A Privacy Preservation Quality of Service (QoS) Model for Data Exposure in Android Smartphone Usage

**DOI:** 10.3390/s21051667

**Published:** 2021-03-01

**Authors:** Anizah Abu Bakar, Manmeet Mahinderjit Singh, Azizul Rahman Mohd Shariff

**Affiliations:** School of Computer Science, Universiti Sains Malaysia, Gelugor 11800, Penang, Malaysia; azizulrahman@usm.my

**Keywords:** smartphone sensors, mobile applications, permissions, risk quantification, privacy exposure, mathematical modelling, data collector, IoT, usage behaviour

## Abstract

An Android smartphone contains built-in and externally downloaded applications that are used for entertainment, finance, navigation, communication, health and fitness, and so on. The behaviour of granting permissions requested by apps might expose the Android smartphone user to privacy risks. The existing works lack a formalized mathematical model that can quantify user and system applications risks. No multifaceted data collector tool can also be used to monitor the collection of user data and the risk posed by each application. A benchmark of the risk level that alerts the user and distinguishes between acceptable and unacceptable risk levels in Android smartphone user does not exist. Hence, to address privacy risk, a formalized privacy model called PRiMo that uses a tree structure and calculus knowledge is proposed. An App-sensor Mobile Data Collector (AMoDaC) is developed and implemented in real life to analyse user data accessed by mobile applications through the permissions granted and the risks involved. A benchmark is proposed by comparing the proposed PRiMo outcome with the existing available testing metrics. The results show that Tools & Utility/Productivity applications posed the highest risk as compared to other categories of applications. Furthermore, 29 users faced low and acceptable risk, while two users faced medium risk. According to the benchmark proposed, users who faced risks below 25% are considered as safe. The effectiveness and accuracy of the proposed work is 96.8%.

## 1. Introduction

The Internet of Things (IoT) is embedded deeply in various domains, such as mobile services, smart homes, enterprise services, smart environments, futuristic, personal and social applications, transportation and logistics, healthcare and utilities [1]. The growth of IoT brings benefits to various fields. However, as the growth of IoT continues, the chances for users’ privacy to be exploited maliciously also increase. Statistics indicate that there are 3.6 billion smartphone users in 2020 and this number is expected to increase to 3.8 billion in the coming year [2]. Smartphone usage is no longer limited to completing important tasks, it is also used for other purposes such as entertainment, finance, navigation, communication, health and fitness, and so on. An Android smartphone contains built-in and externally downloaded applications. As of June 2020, 2.96 million applications were available in the Google Play Store [3].

The users obtain any types of mobile applications that provide the preferred services without being aware of its implications. Mobile application developers request permissions to provide services using resources on Android smartphones. Although the applications can benefit users, the misuse of these resources by applications may result in data leakage and privacy exposure [4].

The research described in this article quantifies the risk of each application, the risk posed by each category of application, and the privacy exposure level of a user in an Android smartphone environment based on their usage behaviour. A model is constructed to determine the risk faced by each Android smartphone user. This study is unique because the model is multifaceted. Most previous works focused on a single attribute or element, while other multifaceted models focused on the specific application instead of covering all applications available in the Google Play Store. This model is different from other existing work because it calculates the risk posed by user applications and system applications that were overlooked by previous works.

The multifaceted model consists of elements, such as the sum of permission levels, number of requested permissions, amount of user data collected, and total size occupied by the application in Android smartphone storage. The quantification of privacy risk using a multifaceted model is vital for several reasons. One of the reasons is to identify the risk level posed by each type of permission requested because it acts as a door for the developers to access users’ data [5,6]. A second reason for quantifying privacy risk using a multifaceted model is to expose the amount of user data collected by each application. This element is compulsory in quantifying privacy risk because normal users do not realize how much data are being accessed and collected by the applications based on their usage behaviour [7,8]. No existing work that has done a privacy model by considering user data size.

A privacy model based on the research explained in this study can be implemented in any field, such as smart homes, which can be used to monitor and collect the subject’s data for analysis purposes and subsequently preserve the subject’s privacy and identity [9]. This study has several significant contributions and provides a new privacy model in quantifying the risk of Android smartphone users to prevent data leakage. Specifically, it answers the following research questions:

(1) What is the solution to quantify and preserve the privacy of users in Android smartphone usage?
(2) What is the system that can perform real-time monitoring of user behaviour and application behaviour in an Android smartphone?
(3) What is the effectiveness of the proposed model compared to other privacy risk models in benchmarking the privacy exposure level of a user?

The focus of this article is on answering the three research questions mentioned. The techniques used in constructing the model produce results that are competitive with existing studies such as LRPdroid [7], PUREdroid [8], Online Social Network privacy score [10], and Identity Theft Assessment and Prediction (ITAP) [11]. The contributions are as follows:

(1) A novel mathematical model to quantify the privacy and risk of the user in an Android smartphone environment is developed. The privacy calculus solution is used to develop the privacy risk model to quantify the risk posed by the applications and the privacy exposure level faced by the Android smartphone user.
(2) New attributes implemented in the development of the model are illustrated using a tree structure. The related attributes used to develop the model are permission-level types, sensor data types, and personal data types.
(3) A multifaceted system is designed to monitor the collection of user data size by accessing the granted permissions and portray the risk posed by the applications in Android smartphone usage. Currently, no multifaceted system is available to monitor the collection of user data size and the risks posed by the applications for each user. Through this system, the user can evaluate the list of permissions requested, the amount of data accessed and collected by an application, and the risks posed by each application.
(4) A benchmark of privacy risk for Android smartphone users is created. Currently, no benchmark of privacy risk has been established for Android smartphone users. By creating this benchmark, users can be aware of the level or range of risks they are facing. They can decide whether they are at risk or vice versa by referring to the benchmark. They can also decide on further actions to protect their privacy based on the benchmark.

The remainder of the study is organized as follows: Section 2 discusses the literature review. Section 3 presents in-depth information on the methodology used in developing the proposed model. Section 4 presents the results. Section 5 presents the discussion related to the privacy model and results. Section 6 presents the conclusion and Section 7 provides the patent information.

## 2. Literature Review

This section presents the context for the study described in this study by giving relevant information on the background and related work. The discussion of related work is fairly extensive to demonstrate that the current study goes well beyond what has been done in previous studies in terms of the applications in the Android smartphone, classification of data sensitivity, Android permission levels that access data, risk-leading elements in mobile application usage, privacy risk model built using calculus and existing privacy models and its features.

### 2.1. Applications in Smartphone

Applications in an Android smartphone can be divided into user and system applications. User applications or mobile applications are defined as self-contained software designed for a mobile device that performs specific tasks for mobile users [12]. User applications can be downloaded externally from any available sources, such as Google Play Store, AppGallery, and APK sites. Users can choose which applications to download according to their preferences. In application store platforms, such as Google Play Store, user applications are grouped into 33 categories of applications according to the main function or types of services provided [13]. Table 1 shows examples of applications for each category of applications.

System applications are pre-installed applications and services provided by the smartphone manufacturers, such as Samsung, Oppo, Huawei, and other Android device manufacturers. The system applications are signed in with the platform signing keys that allow access to the Privileged permission level, which is the extremely high-risk permission. These applications usually act as services that run in the background, monitoring user activities without their consent, and often without their knowledge [14]. Although the manufacturers designed their devices to run using the same Android operating system, these system applications vary according to the manufacturers because they install their self-developed system applications. These applications are unavailable at any external sources because the self-developed system applications work on their manufactured devices only. By doing this, the manufacturers try to provide users with advanced and special features to stand out as the best Android smartphone manufacturer and competitor in the digital market. Table 2 shows several system applications available in Android smartphones such as Samsung [15], Oppo [16], and Huawei [17].

Other examples of common system applications are Calculator, Clock, Messages, and Android System Webview. Both user and system applications may pose risks to users by tracking their activities, collecting their sensitive data, and misuse the data without their consent [18].

### 2.2. Classification of Data Sensitivity

A privacy impact assessment (PIA) addresses the causes of data leakage and privacy breaches [19]. In PIA, the vital step is to identify information that will originate, terminate in, or pass through the IoT-enabled system. An Android smartphone contains both sensitive and non-sensitive information. Each type of data is labelled as not sensitive, lowly sensitive, moderately sensitive, and highly sensitive [20]. However, some data that are labelled as not sensitive or low sensitive, can become highly sensitive when two or more data types are combined [20]. Data in the Android smartphone are collected continuously without considering its sensitivity. All these data, which are personal data and sensor data, are provided by the multiple sensors available in an Android smartphone. Table 3 shows the classification of data.

The data mentioned in Table 3 can be accessed by the developers, intruders, and adversaries when users grant permissions during the application installation. The permission types and levels (exposure levels) will be described in detail in the next subsection because they are related closely to data sensitivity.

### 2.3. Android Permission Levels

Android permissions are the most important element in a mobile application. These permissions are the ones used to request permission from users to access their data. The levels of data sensitivity depend on permission levels. There are 101 permission types available in android permission lists: 36 permissions in the Normal level as listed in Android 9 (API level 28), 26 Dangerous permissions, 29 Signature permissions, and 10 Privileged permissions [14]. Due to the high number of permissions, it is impossible to list all requested permissions. Thus, Android has categorized the permissions into four main levels, namely, Normal, Dangerous, Signature, and Privileged [14]. By doing this, the requested permissions are organized according to these categories. The four permission levels are explained further as follows [14]:

- *Normal*—A permission that grants access to a requesting application with lower risk. The system will automatically provide access to this type of permission to the requesting application without the user’s consent. However, users can review these permissions before installing. For example, ACCESS_NETWORK_STATE, ACCESS_NOTIFICATION_POLICY, BLUETOOTH, EXPAND_STATUS_BAR, SET_ALARM.
- *Dangerous*—This permission level is considered as higher-risk permission because it grants requesting the application the right of entry to users’ sensitive and private data. This permission also allows access to control that particular device which negatively affects the users. Because of the issue, the system may not automatically provide access to the requesting application. For example, ACCESS_COARSE_LOCATION, ACCESS_FINE_LOCATION, CAMERA, ACTIVITY_RECOGNITION, READ_CONTACTS.
- *Signature*—This permission is very risky because it grants permission to the system if the requesting application is signed with a similar certificate as the application that defined the permission. Once the certificates match, the system will automatically permit the permission without notifying the users beforehand. For example, BIND_NFC_SERVICE, BIND_INPUT_METHOD, REQUEST_INSTALL_PACKAGES, BIND_VPN_SERVICES.
- *Privileged*—This permission level is considered extremely risky. This permission is granted only to an application signed with a similar certificate as the application that defined the permission. Developers should avoid using this permission because the Signature permission level is sufficient most of the time. However, developers can still use this permission for certain unavoidable special cases. For example, BATTERY_STATS, CHANGE_CONFIGURATION, CLEAR_APP_CACHE, MANAGE_EXTERNAL_STORAGE, BIND_INCALL_SERVICES.

From the descriptions above, the most common requested permissions in the mobile application are Normal and Dangerous because most applications only request Internet access. The Signature and Privileged are considered as high risk and extremely high-risk permission levels accordingly because they allow the developer to take ownership of the devices. However, the combination of two or more permissions sequent of Normal or Dangerous can become high risk and expose users’ privacy. For instance, if a user downloads Whatsapp, which requires the normal permission for internet access and changing audio settings, using fingerprint hardware, taking photos, reading contacts, and accessing precise location can put users at risk because the accessed data are unencrypted and can be seen in clear text that lead to data leakage [21].

### 2.4. Risk Leading Elements in Mobile Application Usage

Although several factors have affected the efforts for privacy preservation, more specific risk-leading elements need to be described to understand what types of elements lead to privacy exposure. These elements are considered in the proposed work and described further in Table 4.

These risk-leading elements can be implemented into the calculus model to quantify the risks of application and privacy exposure levels for each user. The privacy calculus model is considered as the approach to tackle privacy issues in Android smartphone usage.

### 2.5. Privacy Risk Model Using Calculus

Previous researchers have proposed techniques to preserve the privacy of users. One of the typical and effective theories to quantify privacy risk is the use of the privacy calculus solution [10]. This theory is suitable to be applied in the Android smartphone environment because it can quantify the privacy risk of users effectively and precisely by showing the outcomes in terms of numbers. Users understand more when they see the range of numbers for their privacy level.

The first model to measure privacy scores by quantifying the degree of sensitivity of information and visibility of that piece of information in the network was proposed by Liu and Terzi [10]. The researchers used two attributes or elements in their model, namely, the sensitivity and visibility of information in the network to quantify the privacy faced by the users in the Online Social Network (OSN) environment. However, the model cannot be applied in another domain. The specifically declared category, which is OSN itself, is insufficient to preserve privacy in continuous Android smartphone usage. Furthermore, the attributes declared are insufficient to preserve overall privacy in Android smartphone usage.

Lo et al. [7] developed a user privacy analysis framework called LRPdroid by implementing a privacy calculus solution. The solution is developed to detect information leakage, evaluate user privacy disclosure, and privacy risk assessment. Detection, evaluation, and assessment focused on mobile applications installed on Android smartphones. The model can support users in managing their privacy risk on intended applications. The experiment is done to show the feasibility and practicability of LRPdroid. As a result, Line application is considered as high risk because users disclose their birth date and credit card number when dealing with self-claimed customer service clerk that transmitted data through the Internet. Thus, the user can see the risk posed by applications and are aware of the information disclosure. However, the model focused on quantifying the risk of downloaded applications, which are also known as user applications. The researchers overlooked the fact that any applications installed on an Android smartphone can pose risks, such as system applications pre-installed and ran without the consent of users.

Alshehri et al. [8] proposed a permission usage and risk estimation for Android, environment called PUREDroid that allows users to evaluate the risk level of permissions requested by an application. The elements involved in the development of the model are several benign applications, several malware applications, permissions, and applications. This model is also useful in helping the user to decide whether to grant or deny the requested permissions. It evaluates the malware and benign applications to portray the effectiveness of the proposed assessment model. By using this model, users can be aware of the risks involved in granting permissions to the desired applications. Based on the results, the permissions that execute suspicious activities are assigned to high-risk scores compared to normal activities. However, the model only assessed user applications and overlooked system applications. The model did not reveal the amount of user data being collected by applications, which should be transparent to the users. Without implementing the user data attribute into the model, it is insufficient to protect the users because they may not understand the consequences of exposing the enormous amount of personal data to the applications.

Chih-Chang et al. [11] proposed a framework for estimating the privacy risk scores of mobile applications. They assessed the privacy risk score of open-source applications collected from the Google Play Store. They used access rights and privacy policies of each application to analyze the type of personally identifiable information that is being collected by the applications. However, the work assesses the privacy risk of applications available in the Google Play Store instead of assessing the privacy risk faced by Android smartphone users. Apart from that, the risk assessment does not evaluate system applications that are pre-installed by the manufacturers of Android smartphones. Due to these gaps, the framework is insufficient to protect the privacy of the user in Android smartphone usage.

Based on the previous work done using privacy calculus, the model can produce efficient outcomes. However, privacy issues persist in Android smartphone usage because researchers overlooked several significant elements that need to be considered in the development of a privacy model to preserve the privacy of Android smartphone users. Furthermore, the single-faceted models developed by previous researchers have difficulties in mitigating data leakage in the whole Android smartphone usage. It is in this aspect that the proposed PRiMo model proved to be better than previous studies [7,8,10,11] because it is multifaceted and covers all the categories of applications to preserve the privacy of users in overall Android smartphone usage.

### 2.6. Existing Privacy Models and Features

Several privacy models to preserve users’ privacy have been proposed in previous studies. Although the existing models are good approaches, they lack significant elements when constructing the model, such as multifaceted, permission levels, number of permissions, size of user data being collected, and total size allocated by application in Android smartphone storage. The existing privacy models and their features are summarized in Table 5. The features that are compared between these models are attributes, assessment, environment, and multi/single faceted. These previous works [7,8,10,11] are compared because they have a similarity with the present study. From the existing works, this study found significant elements that have been overlooked in their models.

The existing models are single-faceted because they do not have several significant features. In this study, the features chosen as criteria are sensitivity, visibility, confidentiality, universality, permissions, user data, flexibility component, and compatibility. These features are explained in detail in Section 3.1.

## 3. Proposed Work

The idea is to design a multi-faceted privacy model, which means all the significant features must be embedded because the privacy-preserving model must be secure and comprehensive. This section will discuss in-depth the methods and techniques used in the development of the proposed model. The development of the mathematical model to quantify the privacy risks of a user in Android smartphone usage is explained in detail. The methods start with the classification of data types and extend to the classification of permission levels with assigned values, an overall illustration of risk in mobile application using a tree structure, and the proposed mathematical model to quantify the risk of each application, the risk posed by each category of application and the overall privacy risk faced by a user in an Android smartphone usage accordingly. The two vital elements in developing the model are data types and permission levels. These elements are discussed further.

### 3.1. Features of PRiMo

The features chosen as criteria are sensitivity, visibility, confidentiality, universality, permissions, user data, flexibility component, and compatibility. The features are explained in detail as follows:

- Sensitivity—Sensitivity in Android smartphone usage refers to the exposure level of user data and information.
- Visibility—Visibility is the exposure of data to the developer based on the permissions granted by the device owner through the network.
- Confidentiality—Confidentiality is the protection and preservation of sensitive data.
- Universality—Universality defines the consideration of including the system and user applications in the privacy quantification.
- Permission—Permissions are requested by the developer to obtain authorization rights to access sensor data and personal data collected by an application.
- User data—User data are the data collected from the application usage, including sensor data and personally identifiable data.
- Flexibility—Flexibility is considered in the proposed model to ensure that the model can be implemented in the future when any new types of user data, sensors, and permissions arise.
- Compatibility—Compatibility means the model is capable of quantifying all types of applications, such as APK and non-APK.
- The features of PRiMo are as follows:
- Privacy exposure and application risk quantification level contribute to the sensitivity and visibility features.
- The privacy quantification ensures the confidentiality feature.
- The quantification of risk for system applications and user applications shows that PRiMo has a universality feature.
- The permission feature in PRiMo highlights the actual permission levels requested and the number of permissions, which ensures that applications are being transparent to the user who have the right to know information related to application permissions.
- User data feature is one of the significant features overlooked by previous researchers. The amount of user data collected by an application can be exposed by PRiMo to show the users how much data are being exposed, accessed, and collected by each application because data are sensitive and confidential.
- The flexibility feature is also important in ensuring that the model will fit in the future technologies that may have new sensors, a new declaration of types of user data, and newly declared or requested permissions.
- The compatibility feature is vital in the quantification of privacy exposure level and application risk for APK and non-APK applications because some users might install APK applications from any sources except Google Play Store and manufacturers-declared store. The consequences are, once APK is downloaded from unverified external sources, the APK may contain malicious code and it can access and collect user data tremendously without the consent of users, which will eventually put them at extremely high risk. This type of APKs may also run in the background continuously to collect more than “need to know” information of users.

Thus, the privacy model must be able to protect the overall features of the device to protect and preserve the privacy of users. Existing models have various drawbacks, such as the implementation of single-faceted attributes, limited coverage of categories of mobile application, lack of research on permission types and user data size, non-extensive model, and lack of diversities. PRiMo is the best solution to overcome privacy issues in Android smartphone usage. The proposed work is explained in detail in the next section.

### 3.2. Assignation of Values to Permission Levels

**Figure 1:**
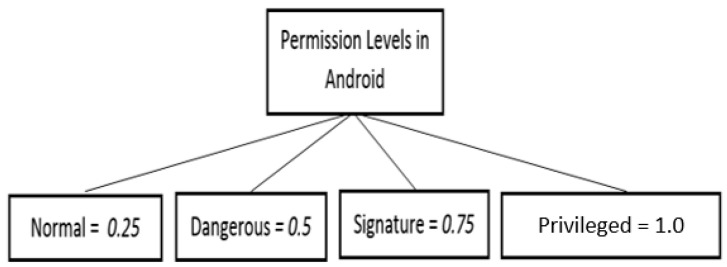
Classification of permission levels and their assigned value.

The developers gain access to user data by requesting permissions. The permissions in the Android platform are grouped into four levels, namely, Normal, Dangerous, Signature, and Privileged. Each permission level is assigned a specific value. Figure 1 shows the classification of permission levels and the assigned values.

Figure 1 shows the four permission levels and their values. For instance, Normal is 0.25 (Level 1), Dangerous is 0.5 (Level 2), Signature is 0.75 (Level 3) and Privileged is 1.0 (Level 4). These values indicate the exposure level of user data sensitivity. Normal permissions are considered as low risk, Dangerous permissions are medium risk, Signature permissions are high risk and Privileged permissions are extremely high risk. Thus, permission levels should be considered when developing a privacy quantification model.

This element is included in the model as one of the privacy risk-leading elements. It is portrayed in the tree structure in the next step, which is to construct the tree structure containing privacy risk-leading elements.

### 3.3. Tree Structure

**Figure 2:**
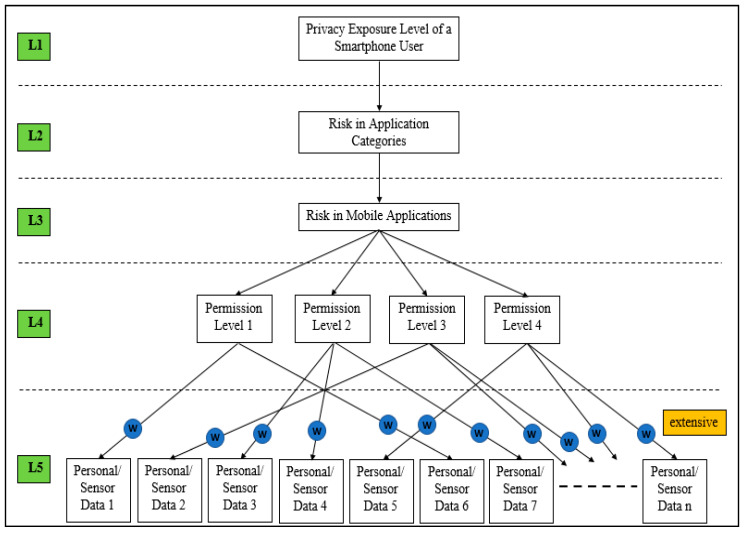
Detailed tree structure.

At this stage, the tree structure is used to clarify and provide a clearer view of elements that lead to privacy risk. Data types and permission levels are illustrated based on graph theory knowledge. Figure 2 shows the tree structure in depth.

Figure 2 shows the five stages in determining the privacy exposure level of a user. The figure shows the overview of the tree structure that portrays the relationship between privacy (L1), risk in each category of application (L2), risk in a mobile application (L3), permission levels (L4), and data types (L5). The user data size, which is labelled as weight, w, is collected and accessed through the permissions granted, which then leads to the risk quantification for each mobile application. Then, the risk posed by each application is summarized to quantify the exposure of user privacy in an Android smartphone environment. The model is developed to be extended to fit advanced and emerging technologies because there might be new data types and sensors in the future. The development of the model is discussed further in the next step.

### 3.4. Proposed PRiMo

To propose a privacy model, the fine-grained data must be declared at the individual application level. Next, all the applications within the category must be quantified. Finally, by doing this, the privacy exposure level for each user in Android smartphone usage will be obtained. The quantification starts with the risk of each installed application including system and user applications, followed by risk quantification for each category of mobile application, and finally the privacy exposure level for each user. The steps are discussed by part for each quantification.

#### 3.4.1. Risk Posed by Each Application

The risk of each application, *R_APP_* involves permission levels, the number of permissions requested, user data size, and total size allocated in Android smartphone storage for the application. The descriptions of notations used in the model are explained as follow:

- *R_APP_* denotes the risk of an application.
- Σ*_PL_* denotes the summation of permission levels in an application.
- *N_P_* denotes the number of permissions requested by an application.
- Σ*W_UD_* denotes the summation of the weight of user data accessed or collected by an application.
- *T_SIZE_* denotes the total size allocated by an application in the Android smartphone storage.

The risk of an individual application model is developed as follows:

(1) $$R_{APP}=\left(\frac{\sum_{PL}}{N_{P}}\right)\;x\left(\frac{{\sum W}_{UD}}{T_{SIZE}}\right).$$

Equation (1) has been completed. The next step is to calculate the risk posed by each category of mobile application, *R_APPCAT_* by extending (1).

#### 3.4.2. Risk Posed by Each Category of Mobile Application

The equation to calculate the risk posed by each category of mobile application, *R_APPCAT_* is extended from (1). The summation of the same type of individual applications’ risk score, RAPP are the risk of each mobile application category, *R_APPCAT_*. The descriptions of notations are as follow:

- *R_APPCAT_* denotes the risk of a mobile application category.
- Σ*_PL_* denotes the summation of permission levels in an application.
- *N_P_* denotes the number of permissions requested by an application.
- Σ*W_UD_* denotes the summation of the weight of user data accessed or collected by an application.
- *T_SIZE_* denotes the total size allocated by an application in Android smartphone storage.

The model to calculate the risk of a mobile application category is as follows:

$$R_{APPCAT}\;=\;R_{APPa}\;+\;R_{APPb}\;+\;\cdots \;+\;R_{APPn}$$

(2) $$R_{APPCAT}=\left(\frac{\sum_{PL_{a}}}{N_{P_{a}}}\right)\;\left(\frac{{\sum W}_{UD_{a}}}{T_{SIZE_{a}}}\right)\;+\;\left(\frac{\sum_{PL_{b}}}{N_{P_{b}}}\right)\;\left(\frac{{\sum W}_{UD_{b}}}{T_{SIZE_{b}}}\right)\;+\;\cdots \;+\;\left(\frac{\sum_{PL_{n}}}{N_{P_{n}}}\right)\;\left(\frac{{\sum W}_{UD_{n}}}{T_{SIZE_{n}}}\right).$$

Equation (2) has been completed. The next step is to calculate the overall privacy risk faced by the user in an Android smartphone environment by extending (2). The model is described further in the next subsection.

#### 3.4.3. Privacy Exposure Level Faced by Each User in Android Smartphone Usage

The model to calculate the privacy exposure level faced by each user in Android smartphone usage, *P_u_* is extended from (2). The summation of risks posed by all available categories of mobile applications in an Android smartphone is *R_APPCAT_*. The multiple *R_APPCAT_* is the privacy risk faced by each user in the Android smartphone environment based on their usage behaviour, *R_APPCAT_*. The descriptions of notations are as follow:

- *P_u_* denotes the privacy risk of a user.
- *R_APPCAT_* denotes the risk of a mobile application category.
- ${\bar{X}}_{P}$ denotes the mean of permissions of applications in an Android smartphone.
- Σ*W_UDi_* denotes the summation of the nth weight of user data accessed or collected by applications.
- Σ*T_SIZEi_* denotes the nth total size allocated by applications in the Android smartphone storage.

The final PRiMo model is developed as follows:

$$P_{U}\;=\;R_{APPCAT1}\;+\;R_{APPCAT2}\;+\;\cdots \;+\;R_{APPCATn}$$

(3) $$P_{U}\;=\;{\bar{X}}_{P}\;\left(\frac{\sum_{i\;=\;1}^{n}W_{UDi}}{\sum_{i\;=\;1}^{n}T_{SIZEi}}\right).$$

Equation (3) shows the final product mathematical model of the Privacy Risk Model, PRiMo. This model includes elements, such as permission levels, number of requested permissions by applications, the weight of user data, and size allocated by applications in Android smartphone storage. These elements are significant in quantifying the privacy risk of Android smartphone users precisely.

The PRiMo is evaluated using the one-Sample *t*-test to prove that the elements or attributes declared in the model are significant and effective in preserving privacy. Next, the evaluation of the model is discussed.

### 3.5. PRiMo Evaluation Using One-Sample t-Test

**Figure 3:**
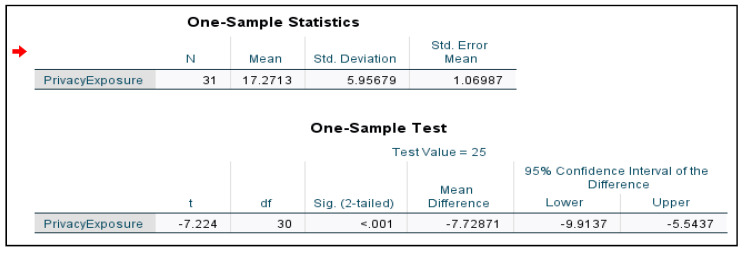
One-Sample *t*-test output.

A one-sample *t*-test was conducted to prove the effectiveness of the proposed model. The model is developed to quantify the privacy exposure level in overall Android smartphone usage. These attributes include the permission levels, number of permissions requested, amount of user data, and the total size of application allocated in the phone storage space are used in the model. The attributes are elements that lead to privacy breaches and data exposure in Android smartphone usage. These parameters correlate to each other to provide accurate and effective quantification. A one-sample *t*-test was performed to prove the significance of the proposed PRiMo model. Because it is a One-sample t-test, the mean of the known sample is referred from previous work [22]. Figure 3 shows the evaluation results of the model.

Figure 3 shows the statistics in the output such as *t*-value, degrees of freedom (df), p-value, and confidence interval value that need to be interpreted by referring to Student *T*-Table to determine the effectiveness. According to Student *T*-Table, the critical value for df 30 is 2.042. For a model to be effective, the *t*-value should be less than the critical value. In this study, the *t*-value, −7.224 < critical value, 2.042. Thus, the findings are significant. Furthermore, the p-value, 0.001 should be less than 0.05. In this study, the findings show that 0.001 < 0.05. Thus, the model is proved to be effective. The confidence interval also should not include 0 between the Lower and Upper value. In this study, the lower value is −9.9137 and the Upper value is −5.5437. The 0 is not included. Thus, the proposed model is proved to be effective in quantifying the privacy exposure level in Android smartphone usage. Next, the proposed AMoDaC is discussed.

### 3.6. Proposed App-Sensors Mobile Data Collector Tool (AMoDaC)

**Figure 4:**
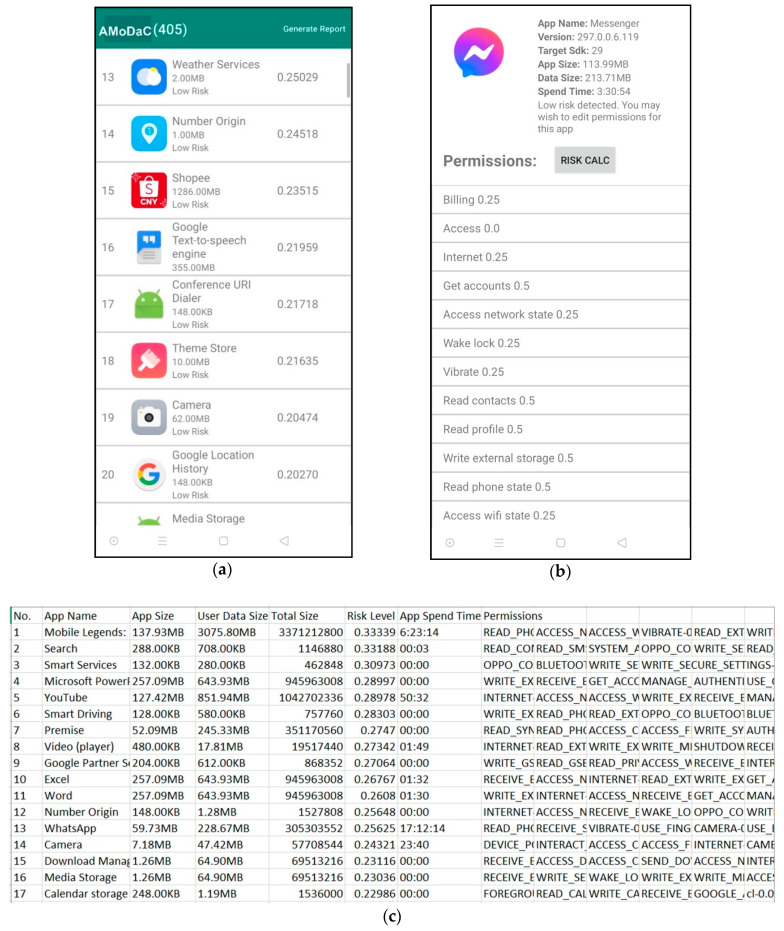
(**a**) Interface of AMoDaC listing all installed applications and their risk values; (**b**) Interface of AMoDaC showing additional information on the specific applications; (**c**) Usage report generated by the user via AMoDaC tool.

This section describes how the data are being collected from the users. We developed an application, called AMoDaC, which acts as a data collector tool that can monitor the usage of the Android smartphone. It can monitor and collect the related information of each user such as application name, application size, the main category of application, user data size, the total size of application allocated in the Android smartphone, lists of permissions and its assigned value, and the privacy risk level. The tool can also display necessary actions that need to be taken based on the risk levels. Figure 4 shows the interfaces and features of AMoDaC.

A range for application risk is assigned based on the assignation of values for each permission level in Section 3.1. The risk range posed by each application is shown in Table 6.

The algorithm is constructed to perform the calculation of risk for each application by referring to the risk range in Table 6. The Algorithm 1 is as follows.
**Algorithm 1:** Calculate risk of each app (permission, number of permissions, user data size, total size)Input: Permission Level (_PL_), Number of permission (N_P_), User Data Size (W_UD_), Total Size (T_SIZE_) Output: Low Risk, Medium Risk, High Risk, Extremely High Risk 1.  $R_{APP}=\left(\frac{\sum_{PL}}{N_{P}}\right)\;\left(\frac{{\sum W}_{UD}}{T_{SIZE}}\right)$ 2. If R_APP_ ≤ 0.25 3. Return Low Risk 4. Else if 0.251 ≤ R_APP_ ≥ 0.5 5. Return Medium Risk 6. Else if 0.51 ≤ R_APP_ ≥ 0.75 7. Return High Risk 8. Else if 0.751 ≤ R_APP_ ≥ 1.0 Return Extremely High Risk

Based on the Algorithm 1, the AMoDaC can quantify risks for each application based on the input retrieved automatically from accessing the components of the usage statistics related to the installed applications. This task can be performed because of the multifaceted feature of the AMoDaC [23]. The outputs that will be displayed in the AMoDaC tool are “Low Risk”, “Medium Risk”, “High Risk” and “Extremely High Risk”. Based on the formula, if the application risk is less than 0.25, it means the application poses a low risk and is considered safe to use. If the application risk is between 0.251 and 0.5, then the application poses a medium risk, which means sensitive and confidential data are being leaked in a small amount. If the application risk is between 0.51 and 0.75, then the application poses a high risk, which means user data are being exposed to a huge amount. Lastly, if the application risk is between 0.751 and 1.0, then the application poses an extremely high risk and user data is being leaked and exposed tremendously.

Based on the risk posed by an application, AMoDaC tool provides recommendations for further action that can be done to reduce the risk, such as rechecking and limiting the requested permissions, uninstalling malicious applications, and resetting the Android smartphone to delete any malicious services running in the background. The effectiveness of the AMoDaC tool is tested in a real environment through experiments and feedback from users through the usability study. The usability study is discussed in the next section.

### 3.7. Proposed Usability Study

The usability study is divided into two types, namely, the usability study on the proposed AMoDaC tool and the usability study on their experience and feedback of users toward risk in Android smartphone usage.

The usability study on the proposed AMoDaC is conducted through a remote experiment where users need to install the AMoDaC, which is available in Google Play Store, and use it as usual. Later, users can share usage reports to perform further analysis regarding their usage and application behaviours that lead to risk and privacy exposure.

Another usability testing is done using Google Forms survey, which was done to obtain the users’ opinion on the proposed tool used, obtain information on the initial awareness of users toward the risk in Android smartphone usage, and their post-opinion towards the risk posed by applications installed on their Android smartphone based on the usage and application behaviours. The obtained information is analysed, and the findings are discussed in Section 4 of this study. Then, the clustering of the category of mobile applications is discussed.

### 3.8. Proposed Category of Mobile Applications

Some enhancements were made to the grouping of applications into main categories. As of July 2020, 36 categories of applications are listed in Google Play Store. Conducting experiments on the 36 categories would be a complex endeavour and thus, the applications were further clustered into 11 main categories, including Augmented Reality, Communication, Education, Entertainment, Finance & Business, Games, Health & Fitness/Medical, Lifestyle, Maps & Navigation, Social Network, and Tools & Utility/Productivity. This main category is one of the vital elements in producing output for this study. Table 7 shows the clustering of categories available in Google Play Store into the specified 11 main categories.

The clustering assists in the quantification of the risks of the mobile application categories. Based on the clustering, the risk posed by categories of mobile applications is discussed in Section 4 in this study.

## 4. Results

This section presents and analyses the results of all the experiments conducted in the study. The experiments were conducted to test the effectiveness of the model. A total of 31 users participated in the experiments. The participants were chosen randomly and have different backgrounds. The results are presented in terms of user vs. application category, application category versus user data size, and user vs. overall privacy risk. The results will be explained in detail in this section.

### 4.1. Results of Users vs. Application Category Risk

**Figure 5:**
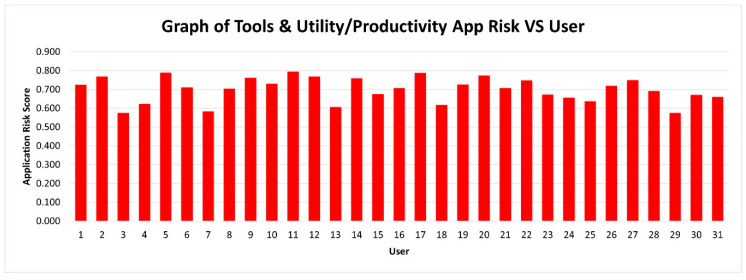
Graph of Tools & Utility/Productivity app risk vs. users.

**Figure 6:**
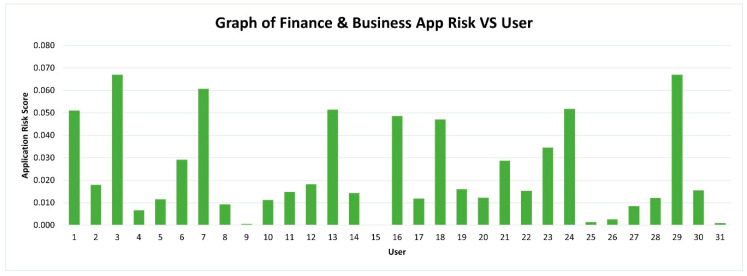
Graph of Finance & Business app risk vs. users.

Some interesting patterns can be observed based on the data collected from 31 users. Previous researchers have identified that utility applications are low risk in contrast to finance-based applications. However, our results indicate the opposite. The findings are presented in Figure 5 and Figure 6.

Among the 11 main categories, 10 categories pose low risk, except for Tools & Utility/Productivity. The graph for Tools & Utility/Productivity is presented specifically in this article to show that the risks posed by this category are high and extremely high as compared to others. Based on the scale, the risk level of Tools & Utility/Productivity for some users are between 0.51–0.75, which is considered as High Risk, and some users even exceeded 0.751, which is considered as Extremely High Risk. Besides, the system applications are mostly under the Tools & Utility/Productivity category. Thus, the number of Tools & Utility/Productivity applications installed in Android smartphones, the permission levels requested, user data amount collected, and the total space allocated in storage are high. Previous studies described finance-related apps as extremely risky [24,25,26]. However, this study proved that finance-related apps are not extremely risky because the risk is between 0–0.25, which is low. This result is because this type of application mostly requests a necessary number of permissions and most permissions are only needed for its functionality. This finding is in contrast to Tools & Utility/Productivity apps such as CamScanner, Calendar, Microsoft Office, Notes, etc. These applications request a more dangerous level of permissions, high numbers of permissions, and a high amount of user data.

### 4.2. Results of User Data Sizes vs. Users

**Figure 7:**
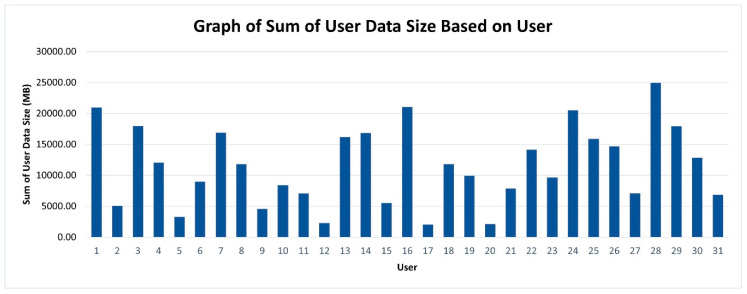
Graph of the sum of user data size vs. users.

Previous researchers have overlooked the user data attribute. User data size should be embedded in the model because it determines the risk level faced by the device owner. It consists of sensitive and non-sensitive personal data and sensor data that could be used to identify a person. The user data sizes vs. users analysis are important to portray the amount of data collected by the applications in the whole usage of the Android smartphone because the high collection of user data might lead to data leakage and privacy breaches. Figure 7 shows the graph of user data sizes vs. users.

Figure 7 shows that the collections of user data are considered high for 26 users because it exceeded 5000 MB worth of data, which might contain highly sensitive data. The user data exposure is very high. The results of privacy exposure level risk vs. user will be discussed further in the next subsection.

### 4.3. Results of Privacy Exposure Level for Each User in Android Smartphone Usage

**Figure 8:**
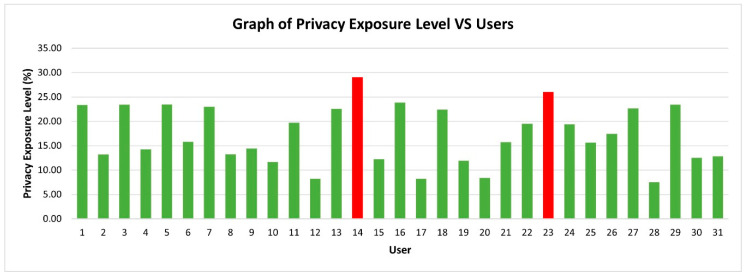
Privacy exposure level vs. users.

An analysis was conducted for the privacy risk faced by each user in an Android smartphone environment based on their usage behaviour. Figure 8 shows the graph of the total risk score of each user in Android smartphone usage.

Figure 8 shows that 29 users are at low risk, which means little data are being exposed and the risk is acceptable at the time that the experiment was conducted. However, nine users might face medium risk if they did not perform early preventive measures. Meanwhile, 2 users out of 31 users are at medium risk, which means their data are being exposed and privacy breaches are occurring. The privacy risk level is determined based on the scale shown in Table 8.

Table 8 shows that if a user faced a low risk in Android smartphone usage, it is acceptable, and they can continue using their device without worries. When the user faced a medium risk, they must check on the applications in terms of permission levels requested, permissions granted and collection of user data size as these elements contribute to the privacy risk level. The results show that 50% of user data is being accessed and exposed to these two users. No users that faced high risk and extremely high-risk level, which is above 50.1%. These results are a good sign of Android smartphone usage in preserving the privacy of users and mitigating data leakage. This result is also due to the Google Play Store filtering most of the malicious applications although several malicious applications bypassed the review process. Another reason is that Android smartphones nowadays have a built-in phone manager that can be used to clean malicious applications or kill applications running in the background without users’ consent. If the privacy exposure level is high (between 50.1% to 75%), then a maximum of 75% of user data is being accessed and exposed. Finally, if a user exceeded 75.1% of the privacy exposure level, they are at extremely high risk. At this stage, the Android smartphone might be controlled remotely without their consent. Thus, users are advised to perform a factory reset on an Android smartphone to eliminate any malicious files and applications.

## 5. Discussion

The main findings of this work suggest that the privacy risk model can be applied to multiple categories of Android smartphone applications. Android smartphones are shown to have user applications and system applications. When quantifying the privacy of the user in Android smartphone usage, both types of applications should be considered because system applications also request the same permission levels as user applications. Moreover, system applications request more high-risk permissions. Although system applications are signed with the manufacturer’s certificate [27], they still pose risks to the users and might lead to data leakage.

In terms of developing the privacy risk model, calculus knowledge should be used because it provides a precise and accurate privacy risk score [28,29]. Users can understand the risks they face based on their usage behaviour. The elements that should be considered in constructing the model are permission levels, number of permissions, user data size, and total space allocated by the application in the Android smartphone storage. The acceptance of the privacy risk score becomes stronger when a multifaceted model is applied to Android smartphone use that consists of diverse applications. Overall, the findings of this study indicate that a multifaceted privacy risk model can quantify the risk efficiently and accurately. The combinations of these elements provide accurate privacy risk.

In terms of practicality, a social theory, such as the usage behaviour of the user, is considered in this study. The relationship between usage behaviour and elements considered in quantifying privacy risk enhances the efficiency of the privacy risk model, which is tested on the different backgrounds of users. The acceptance of the proposed work becomes stronger when a multifaceted model is applied to preserve privacy in Android smartphone usage. In brief, users have a high belief towards the proposed work and the feedback obtained from the application usage, and the proposed model and tool have a significant contribution. Thus, the elements declared in this study are useful in the privacy field.

According to the privacy risk scale, if a user’s privacy risk is between 0–25%, it is considered as low risk. Users can accept the risk and continue using an Android smartphone as usual. If the privacy risk is between 25.1–50%, it is considered as medium risk. The user is at the start of the danger zone because, in this risk range, sensitive data are being monitored, collected, configured, and might also be manipulated. At this level, the user is advised to re-check the permissions granted and the user data amount accessed and collected by the applications. If the privacy risk level is between 50.1–75%, it is considered as a high risk. It is can be stated that 75% of the whole Android smartphone usage is being controlled by intruders including gaining access to data and performing manipulations on the information. At this level, the user is advised to uninstall the risky applications and search for alternative applications. The highest risk level is between 75.1–100%, which is extremely high risk. 

In terms of the effectiveness of the model, the risks posed by each application, category of application, and privacy exposure level are quantified. We quantify each application because it poses a different risk level although they are in the same category of application. For instance, WhatsApp and Messenger are examples of applications under the Communication category. However, these two applications pose a different risk level. For example, according to User 1, WhatsApp poses more risk than the Messenger application. We also quantify the risk posed by each category of application on an Android smartphone because normal users tend to think that finance-related applications are riskier compared to other categories of applications [30]. However, results obtained show that Finance & Business applications are not risky. Instead, Tool & Utility/Productivity applications pose an extremely high-risk level because of the highly requested permission levels, the high number of permissions, and access to a high amount of user data. The privacy exposure level for each user is also quantified to show that each user faces different privacy exposure levels although they install the same application because the risk and privacy exposure level depend on the usage behaviour of the user.

Based on the results obtained, the proposed work is benchmarked with existing works [7,8,10,11] to show the comparison of the features and effectiveness of the proposed work with the existing works and provide knowledge to the user on the risks that can be neglected and risks that need to be avoided to preserve privacy. Intruders might have full access to the Android smartphone, and it might be accessed remotely without the consent of the user. At this level, it is highly recommended that the user performs a factory reset on the Android smartphone to uninstall all applications to preserve privacy and prevent data leakage.

## 6. Conclusions

The objectives of this study were achieved. The first objective was to formalize a mathematical equation using a tree structure and propose a mathematical model designed using the privacy calculus solution that will preserve the privacy of users in the Android smartphone environment. As an achievement, a tree structure is constructed and a mathematical model called PRiMo developed. The second objective was to design a multifaceted system that can perform real-time monitoring and collection of information on user behaviour and application behaviour in an Android smartphone environment. As an achievement, an app-sensor mobile data collector, called AMoDaC, was designed and developed. The final objective was to benchmark the proposed privacy risk model outcome with the existing available testing metrics. As an achievement, the proposed works are evaluated and a benchmark with existing metrics is completed.

The study also has contributions in terms of applicability and scientifically. For applicability, the proposed metric within AMoDaC can be used to monitor any users’ or organization employees’ Android smartphone usage. The use of this tool will reduce the probability of being a victim of any form of attacks that exploit Android smartphones. Scientifically, the term coined as privacy risk is suitable when dealing with users preserving their risk in Android smartphone usage. Optimisation in data structure and size can be enhanced by using the tree structure in mathematical modelling. Another important knowledge is the essence of the multifaceted model in any privacy design. These features are deemed to be important as input in any application tapping on privacy by design.

As a result, it shows that 50% of user data is being accessed and exposed to these two users. No users that faced high risk and extremely high-risk level, which is above 50.1%. These results are a good sign of Android smartphone usage in preserving the privacy of users and mitigating data leakage. This result is also due to the Google Play Store filtering most of the malicious applications although several malicious applications bypassed the review process. Another reason is that Android smartphones nowadays have a built-in phone manager that can be used to clean malicious applications or kill applications running in the background without users’ consent. If the privacy exposure level is high (between 50.1% to 75%), then a maximum of 75% of user data is being accessed and exposed. Finally, if a user exceeded 75.1% of the privacy exposure level, they are at extremely high risk. At this stage, the Android smartphone might be controlled remotely without their consent. Thus, users are advised to perform a factory reset on an Android smartphone to eliminate any malicious files and applications.

In conclusion, the privacy-preserving model such as PRiMo and AMoDaC should be mandatory to protect users’ privacy in the Android smartphone environment based on their usage behaviour. The privacy model proposed in this research is an adaptive risk management type. The importance to add more behavioural based characteristics derived from user behaviour especially as the victim actor of cybercrime threats is crucial. It is essential for more research towards understanding the Tactic, Techniques and Procedures (TTPs) in any cyber threats occurring in smartphone environment and its quantification with privacy risk model such as AMoDaC is explored further. Thus, in future, the adaptive privacy risk model proposed here could stand apart of a risk assessment module in any cyber threat intelligences in tackling security attacks such as Advanced Persistent Threat (APT).

## 7. Patents

Intellectual Property (IP) is registered for both PRiMo and AMoDaC via the Intellectual Property Corporation of Malaysia. The IP number for PRiMo is LY2020003916.

## Figures and Tables

**Table 1:** Categories of applications and their examples [13].

Category of Application	Example of Application
Art & Design	Canva, Logo Esport Maker
Auto & Vehicles	Maxim, 70mai
Beauty	Sweet Snap Live Filter, Beauty Makeup Editor
Books & Reference	MySPRSemak, Innovel
Business	ZOOM Cloud Meetings, Google Meet
Comics	WEBTOON, MangaToon
Communication	Whatsapp Messenger, Telegram
Dating	Hawaya, Borak
Education	Cake, Google Classroom
Entertainment	iQIYI Video, Viu
Events	Blackpink Call Me, Best Music Ringtone for TikTok
Finance	Touch ‘n Go eWallet, Maybank2u MY
Food & Drink	Foodpanda, McDelivery
Games	Mobile Legends, PUBG
Health & Fitness	MySejahtera, Mi Fit
House & Home	PropertyGuru Malaysia, SPEEDHOME
Libraries & Demo	V380 Pro, PUB Gfx Tool
Lifestyle	Pinterest, MY FamilyMart
Maps & Navigation	Grab, Waze
Medical	My AIA, Pregnancy Tracker
Music & Audio	Spotify, JOOX Music
News & Magazines	Twitter, Harian Metro Mobile
Parenting	My Family, Be Closer
Personalization	Fonts, Fantasy Color Call
Photography	InShot, PicsArt
Productivity	CamScanner, Microsoft OneNote
Shopping	Shopee, Lazada
Social	Instagram, Facebook
Sports	Premier League, Live Football TV HD
Tools	SHAREit, Google Translate
Travel & Local	Agoda, SOCAR
Video Players & Editors	TikTok, YouTube
Weather	Rain Alarm, Weather

**Table 2:** Built-in system applications.

Manufacturer	System Application
Samsung	Bixby
	Samsung Pay
	Samsung Health
	Samsung Cloud
Oppo	Oppo Share
	Oppo Relax
	HeyTap Cloud
	Soloop
Huawei	Huawei Health
	Huawei Cloud
	Huawei HiVision

**Table 3:** Classification of data sensitivity [20].

Type	Parameter	Sensitivity
Account creation	Login	Low sensitive
	Password	Highly sensitive
	Name, Address, Phone number	Highly sensitive
	Age	Low sensitive
	Gender	Low sensitive
	Account number	Highly sensitive
Attachment to a smartphone application	Account number	Moderately sensitive
	Serial number	Low sensitive
	Settings and configurations	Low sensitive/Moderately sensitive (depending on attributes)
Daily usage	Image	Highly sensitive
	Microphone data (voice recordings)	Highly sensitive
	Transcribed microphone data	Highly sensitive

**Table 4:** Risk-leading elements and their effects.

Elements	Effect
Permissions	Highly sensitive and confidential data can be accessed and may be exposed without the consent of users.
Number of permissions	The higher number of requested permissions leads to more access to the collection of user data.
User data	User data can be easily stolen, manipulated, and misused for personal benefit by attackers.

**Table 5:** Existing works and their features.

Author(s)	Model	Features	Assessment	Environment	Multi/Single Faceted
[8]	PUREDroid	No. of benign apps; no. of malware apps; permissions; user applications	Risk level of permissions requested by an app	Applicable to user apps	Single-faceted
[7]	LRPdroid	User apps	Detect information leakage and privacy risk	Applicable to user apps	Single-faceted
[10]	Privacy Score	Data sensitivity; data visibility	Privacy score	Applicable in Online Social Network (OSN)	Single-faceted
[11]	Identity Theft Assessment and Prediction (ITAP)	Permissions; open-source apps; sensitivity	Identity theft assessment	Applicable to open-source Android mobile apps	Single-faceted

**Table 6:** Range of risk level indication.

Risk Range	Risk Level
0–0.25	Low Risk
0.251–0.5	Medium Risk
0.51–0.75	High Risk
0.751–1.0	Extremely High Risk

**Table 7:** Clustering of mobile applications into main categories.

Main Category	Application in Play Store
Augmented Reality	Augmented Reality
Communication	Communication
Education	Parenting, News & Magazines, Education, Books & References
Entertainment	Sports, Music & Audio, Entertainment, Comics
Finance & Business	Shopping, Finance, Business
Games	Games
Health & Fitness/Medical	Wear OS, Medical, Health & Fitness
Lifestyle	Lifestyle, House & Home, Food & Drink, Events, Daydream, Dating, Beauty, Auto & Vehicles
Maps & Navigation	Travel & Local, Maps & Navigation
Social Networking	Social
Tools & Utility/Productivity	Video Players & Editors, Tools, Productivity, Libraries & Demo, Photography, Personalization, Art & Design

**Table 8:** Range of privacy risk level.

Range (%)	Privacy Risk Level
0–25	Low Risk
25.1–50	Medium Risk
50.1–75	High Risk
75.1–100	Extremely High Risk

## Data Availability

The data presented in this study are available on request from the corresponding author. The data are not publicly available due to privacy reason.
